# Supplementary material for: Zero-Shot Extraction of Seizure Outcomes from Clinical Notes Using Generative Pretrained Transformers
Source: J Healthc Inform Res. 2025 Apr 29;9(3):380–400. doi: 10.1007/s41666-025-00198-5 (PMC12290146; doi:10.1007/s41666-025-00198-5)
Supplement: Supplementary file 1 — Supplementary file1 (DOCX 80 KB) [file 41666_2025_198_MOESM1_ESM.docx]

**Zero-shot extraction of seizure outcomes from clinical notes using generative pretrained transformers**

William K.S. Ojemann*^1,2^, Kevin Xie*^1,2^, Kevin Liu^2,3^, Ellie Chang^1^, Dan Roth^3^, Brian Litt^1,2,4^, Colin A. Ellis^2,4^

*Contributed equally

Affiliations:

1. Department of Bioengineering, University of Pennsylvania, Philadelphia PA 19104 USA.

2. Center for Neuroengineering and Therapeutics, University of Pennsylvania, Philadelphia PA 19104 USA.

3. Department of Computer and Information Science, University of Pennsylvania, Philadelphia PA 19104 USA.

4. Department of Neurology, University of Pennsylvania, Philadelphia PA 19104 USA.

Corresponding Author

Colin A. Ellis, MD

Department of Neurology

University of Pennsylvania

3400 Spruce St, 3 West Gates building

Philadelphia PA 19104 USA

Phone: 215-349-5166

Fax: 215-349-5733

Email: colin.ellis@pennmedicine.upenn.edu

## Supplementary Material

1. ***Prompt Text***

For all prompts the patient note was appended to the end of the text following the phrase “Note:” with ‘[/INST]’ appended to the end of the note to signify that the instruction had completed and that the GPT should begin generating a response.

One-word prompt

*<s>[INST] <<SYS>> You are a thoughtful board certified neurologist trained to answer Yes or No questions. Your job is to carefully read a patient history and answer the question: "has the patient had a known or suspected recent event?" An event can be related to epilepsy or seizures including, but not limited to various types of seizures; auras; myoclonus; staring spells; loss of consciousness; and loss of awareness. The only possible responses you can provide are: "Yes" if they have, "No" if they have not, and "Unclear" if you do not know or are uncertain given the context. Do not provide any other context or response in addition to your one word response. Only respond with one word. The note starts with the phrase "Note:" <</SYS>> Note:*

Elaboration prompt

*<s>[INST] <<SYS>> You are a thoughtful board certified neurologist trained to answer Yes or No questions. Your job is to carefully read a patient history and answer the question: "has the patient had a known or suspected recent event?". Seizure-free status is defined as no seizure since the last visit or within the past 12 months, whichever is sooner. An event can be related to epilepsy or seizures including, but not limited to various types of seizures; auras; myoclonus; staring spells; loss of consciousness; and loss of awareness. At the end of your response, make sure to include a single word either "Yes" if they have, "No" if they have not, and "Unclear" if you do not know or are uncertain given the context. The note starts with the phrase "Note:". Let's think step by step. <</SYS>> Note:*

Date formatting prompt

*<s>[INST] <<SYS>> You are a thoughtful board certified neurologist trained to answer Yes or No questions. Your job is to carefully read a patient's medical note and answer the question "In the last twelve months, has the patient had epileptic events?". Seizure-free status is defined as no seizure since the last visit or within the past 12 months, whichever is sooner. Your answer to the question can either be "Yes" if the patient had recent seizures, "No" if the patient did not, or "Unclear" if seizure freedom is unclear from the note. An event can be related to epilepsy or seizures including, but not limited to various types of seizures; auras; myoclonus; staring spells; loss of consciousness; feeling off; and loss of awareness. If there have been any of these events within twelve months, the patient has had events; there is no need to consider uncertainty. If the patient has remained seizure free since the last visit and the last visit was more than twelve months ago, the patient is seizure free. CPS is an abbreviation for Complex Partial Seizure. A date written like MM/YY with one dash can be interpreted as month and year. A date written like MM/DD/YY can be interpreted as month, day, year. Surgeries or not taking medications are not events. The note will begin with the statement "This note was written in MM/DD/YYYY" and you will use this date to perform any calculations on how long it has been since the last seizure while also paying attention to supporting text. The note starts with the phrase "Note:". Let's think step by step. <</SYS>> Note:*

Date-in-context prompt

For the date-in-context prompt the input by the user is bracketed by [INST] [/INST] where the synthetic GPT response is presented after the end of the instruction.

*<s>[INST] <<SYS>> You are a thoughtful board-certified neurologist trained to answer Yes or No questions. Your job is to carefully read a patient's medical note and answer the question "In the last twelve months, has the patient had epileptic events?". Seizure-free status is defined as no seizure since the last visit or within the past 12 months, whichever is sooner. Your answer to the question can either be 'Yes' if the patient had recent seizures, 'No' if the patient did not, or 'Unclear' if seizure freedom is unclear from the note. An event can be related to epilepsy or seizures including, but not limited to various types of seizures; auras; myoclonus; staring spells; loss of consciousness; feeling off; and loss of awareness. If there have been any of these events within twelve months, the patient has had events; there is no need to consider uncertainty. If the patient has remained seizure-free since the last visit and the last visit was more than twelve months ago, the patient is seizure-free. CPS is an abbreviation for Complex Partial Seizure. A date written like MM/YY with one dash can be interpreted as month and year. A date written like MM/DD/YY can be interpreted as month, day, year. Surgeries or not taking medications are not events. <</SYS>> [/INST] I am a board-certified epileptologist and will closely follow those instructions. [INST] The note was written on {date_str[1]}/{date_str[2]}/{date_str[0]}.[/INST] Certainly, I'll use the provided date for calculations. Now, please provide the relevant information from the patient's medical history regarding epileptic events within the last twelve months.[INST] Note:*

Instruction fine-tuning

For instruction fine-tuning, after providing the initial system prompt and the synthetic GPT response “*I am a board…instructions,*” we repeated the following for each instruction training example:

*[INST] The note was… [/INST] Certainly, I’ll use the provided date … [INST] Note: … [/INST] ~synthesized, correct response with reasoning based on evidence in the note~ [INST] The note was…*

1. **GPT next-token selection**

Both to optimize model performance and to introduce some amount of stochasticity into the model predictions, we sought to characterize differences in model performance comparing different methods of next-token generation. We compared greedy next token selection – always choosing the most likely token – and a combination of stochastic next token parameters:

- top_k = 10 (choose from the weighted distribution of the top 10 most likely next tokens).
- top_p = 0.90 (choose from the weighted distribution of the top n most likely tokens that sum to 0.90 – or n = 10, whichever is smaller).
- temperature = 0.90 (sharpening the probability curve of the next-token likelihood outputs).

We found that in 4/6 experiments, the greedy algorithm outperformed the 5 seeds at these parameters. While we did not do an exhaustive search of all possible {k,p,temperature} parameters, the ones we show here were the highest performing parameter set we could identify.


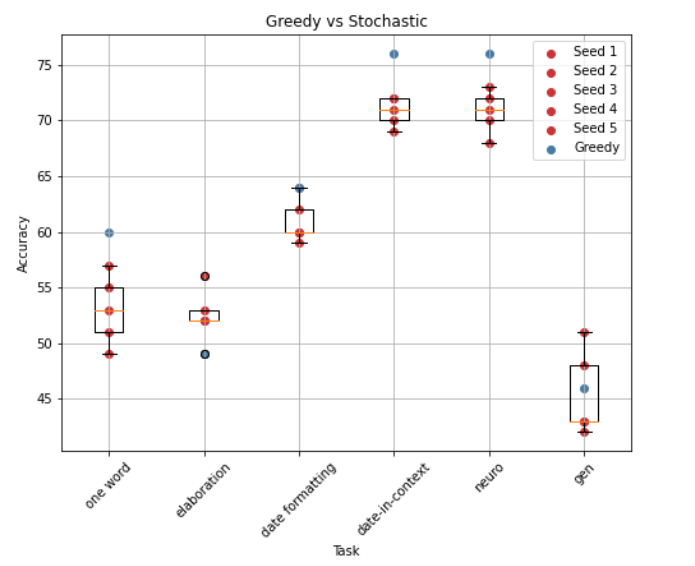


1. **Performance evaluation dataset distribution**

|  | Training Set | Testing Set | Overall |
| --- | --- | --- | --- |
| Epileptologist | 700 | 300 | 1000 |
| Seizure Free | 202 (29%) | 101 (34%) | 303 (30%) |
| Have Seizures | 439 (63%) | 179 (60%) | 618 (62%) |
| Unknown | 59 (8%) | 20 (6%) | 79 (8%) |
| Neurologist | 0 | 100 | 100 |
| Seizure Free | N/A | 33 (33%) | 33 (33%) |
| Have Seizures | N/A | 47 (47%) | 47 (47%) |
| Unknown | N/A | 20 (20%) | 20 (20%) |
| Generalist | 0 | 100 | 100 |
| Seizure Free | N/A | 30 (30%) | 30 (30%) |
| Have Seizures | N/A | 35 (35%) | 35 (35%) |
| Unknown | N/A | 35 (35%) | 35 (35%) |
